# Neuroimaging Characteristics as Diagnostic Tools in Joubert Syndrome and Related Disorders: A Case Report and Literature Review

**DOI:** 10.7759/cureus.69872

**Published:** 2024-09-21

**Authors:** Israa Alhashimi, Sohaib Zoghoul, Sondos K Khalil, Zahra B Yousif, Ammar Jumah, Yaman Alkailani

**Affiliations:** 1 Radiology, Hamad Medical Corporation, Doha, QAT; 2 Internal Medicine, Hamad Medical Corporation, Doha, QAT; 3 Neurology, Henry Ford Health System, Detroit, USA; 4 Neuroradiology Section, Neuroscience Institute, Hamad Medical Corporation, Doha, QAT

**Keywords:** ciliopathies, joubert anomaly, joubert syndrome, joubert syndrome-related disorders (jsrds), molar tooth midbrain-hindbrain malformation, molar tooth sign

## Abstract

Joubert syndrome and related disorders (JSRD) present diagnostic challenges due to their varied clinical features. Neuroimaging, particularly MRI and CT, is critical for identifying the distinctive "molar tooth sign" and other neuroanatomical abnormalities. This case report and literature review emphasize the role of neuroimaging in diagnosing JSRD. Our search targeted pediatric cases with terms like "Joubert anomaly" and "diagnostic imaging." Key findings include cerebellar vermal agenesis, ataxia, developmental delay, and oculomotor apraxia. Cognitive impairment ranges widely, complicating assessment. CT scans reveal dysplastic or absent cerebellar vermis, while MRI shows the characteristic "molar tooth" sign and additional abnormalities such as malformed cerebellar peduncles and enlarged posterior fossa. Accurate diagnosis of JSRD depends on correlating clinical symptoms with specific radiological findings. A multidisciplinary approach is vital for managing this complex disorder.

## Introduction

Joubert anomaly, alternatively termed vermian aplasia or molar tooth midbrain-hindbrain malformation, is an autosomal recessive disorder characterized by varying degrees of absence of the cerebellar vermis. When associated with anomalies in the kidneys, liver, and/or eyes, the condition is referred to as Joubert syndrome and related disorders (JSRD) [1]. This complex neurological condition has become a subject of profound interest within the field of neuroradiology due to its distinct radiological features, its wide-ranging phenotypic diversity, and the evolving paradigms of its classification [2]. The first case of Joubert syndrome was described in 1969 by pediatric neurologist Dr. Marie Joubert, in a child presenting with hyperpnea, abnormal eye movements, intellectual disability, and ataxia. Neuroimaging highlighted the core abnormalities of agenesis of the cerebellar vermis, abnormal 4th ventricle, and thin pons. Several years later, Joubert syndrome was introduced as a distinct syndrome [3,4]. In this article, we present a case of an 88-day-old infant who presented with multiple congenital anomalies. Subsequent non-contrast multiplanar MRI of the head revealed a spectrum of profound radiological abnormalities that were consistent with JSRD. JS is often missed clinically and radiologically if enough attention is not paid to its subtle and variable clinical presentation. The objective of this study is to clarify the clinical and radiological features of JS and to increase awareness of this rare congenital malformation.

## Case presentation

**Figure 1:**
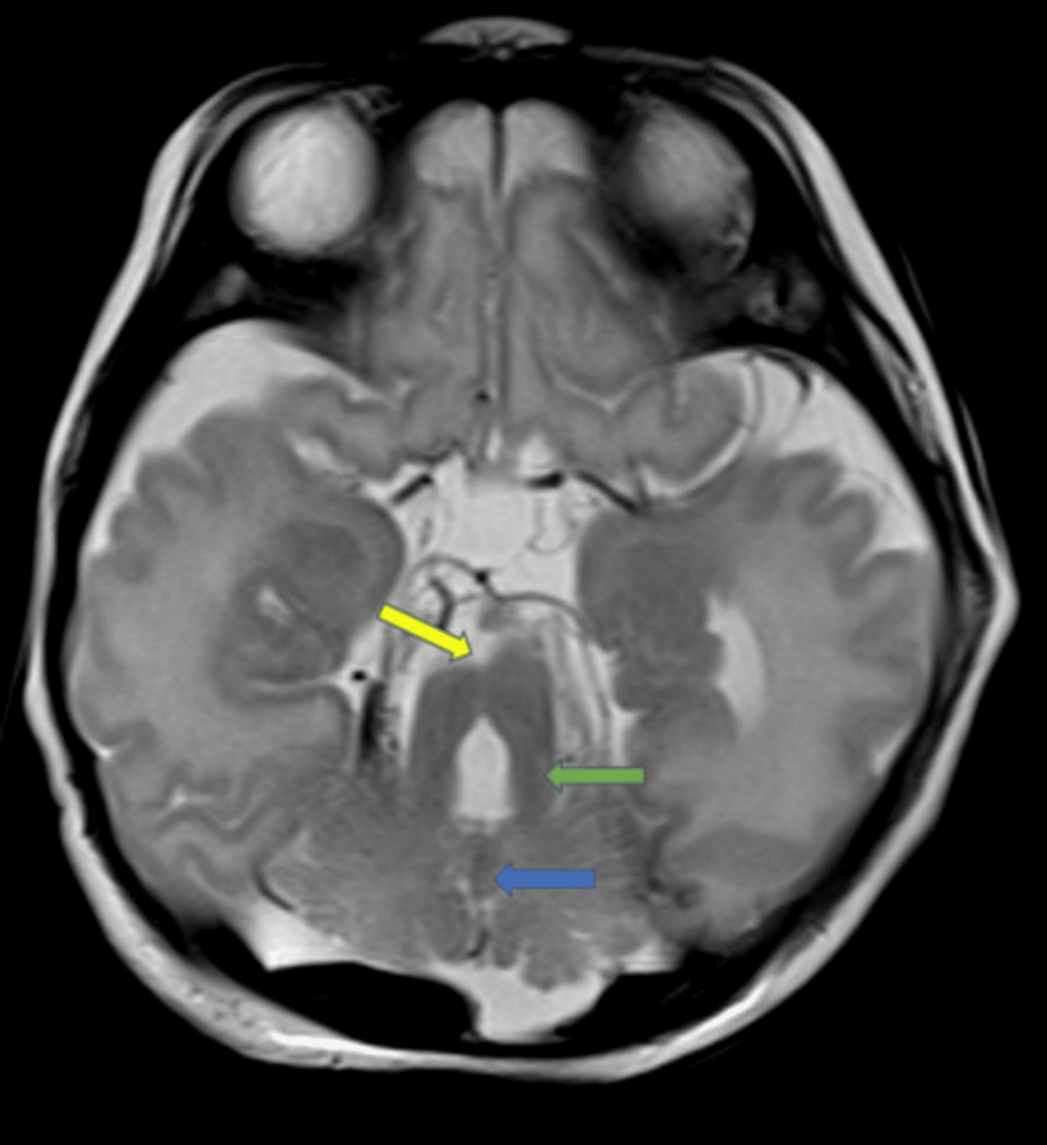
Axial T2W brain MRI shows molar tooth sign (deep interpeduncular fossa (yellow arrow), elongated thick superior cerebellar peduncles (green arrow), and hypoplastic vermis (blue arrow).

**Figure 2:**
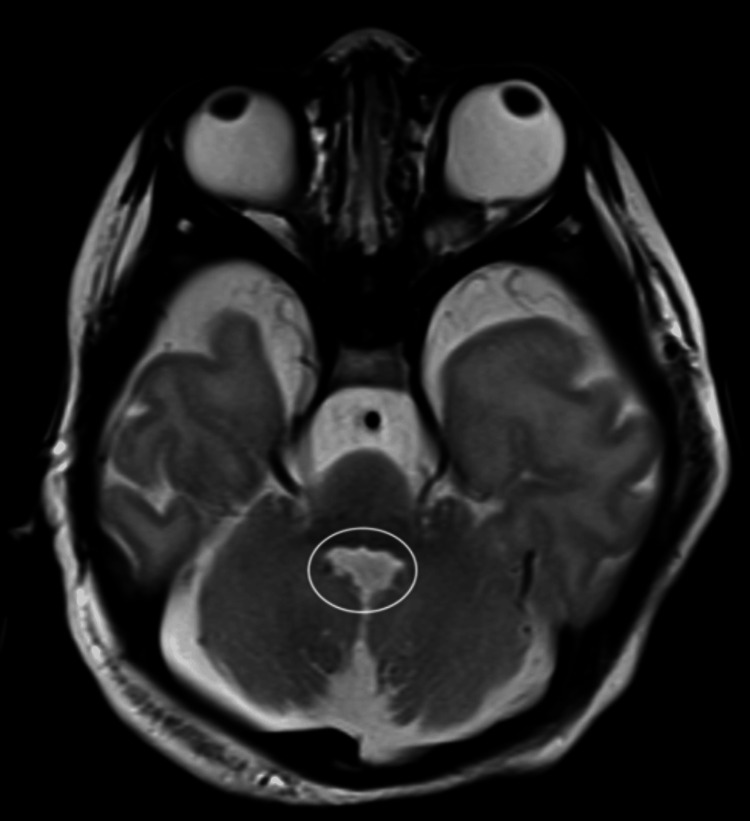
Axial T2W brain MRI: bat-wing sign (absent of vermis with opposed cerebellar hemisphere giving this fourth ventricle morphology (white circle)).

**Figure 3:**
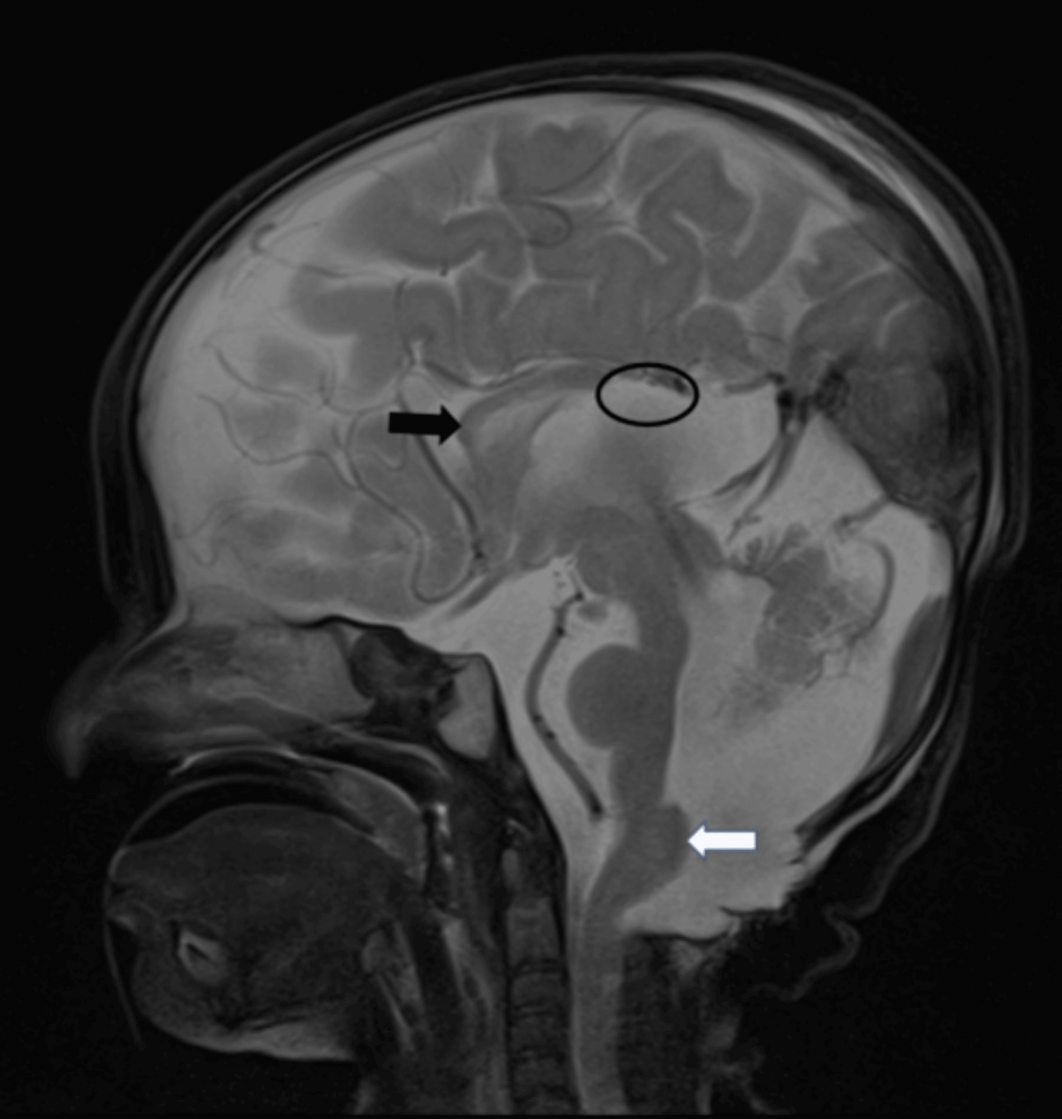
Sagittal T2W MRI shows incomplete absence of the corpus callosum isthmus and splenium (black circle) with residual corpus callosum genu and body (black arrow). The image shows hypertrophied clava or lower brain stem (white arrow).

**Figure 4:**
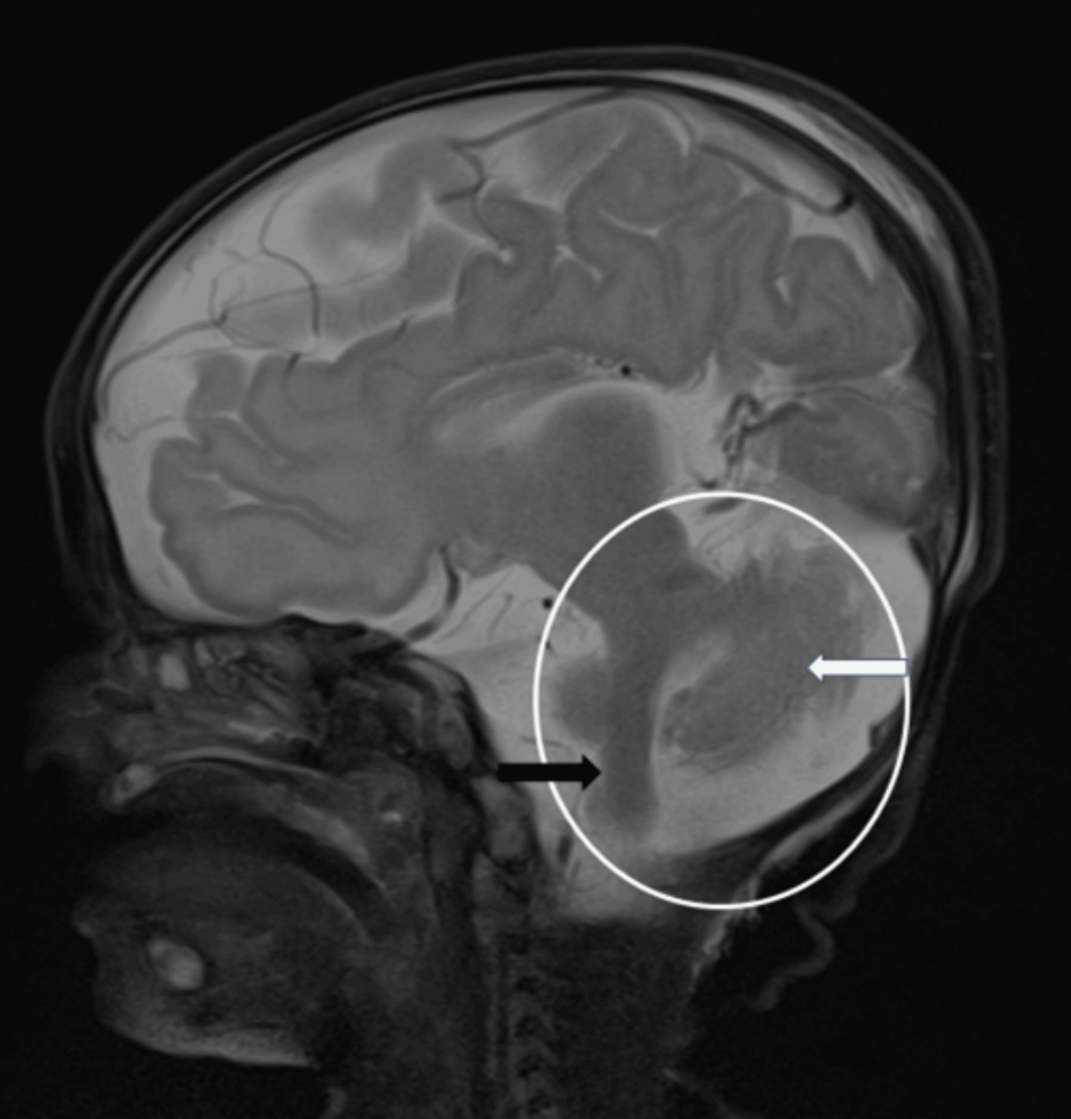
Sagittal T2W brain MRI: Shepherd’s crook sign (white circle) where the shaft is made by the brain stem and pons (black arrow) and the arc of crook made up of the abnormal superior cerebellar peduncle and hemispheres (white arrow).

**Figure 5:**
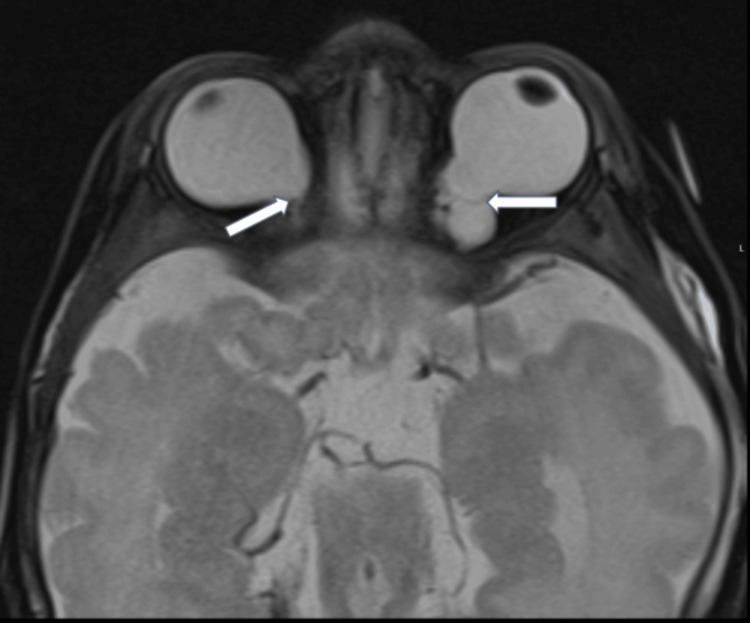
Axial T2W orbital MRI: bilateral coloboma (focal discontinuity in the posterior structures of the eye (white arrows)).

In this case, an 88-day-old infant, born at 38 weeks, presented with multiple congenital anomalies including cleft lip and palate, bilateral iris coloboma, and suspected Dandy-Walker syndrome. The initial skull ultrasound (US) indicated features suggestive of Dandy-Walker malformation, but due to suboptimal image quality, further evaluation was required. Subsequent non-contrast multiplanar MRI of the head revealed a spectrum of profound radiological abnormalities. These included thickened elongated superior cerebellar peduncles (Figure 1), an abnormal midbrain-to-pons ratio, a characteristic "batwing" appearance of the fourth ventricle (Figure 2), aplastic cerebellar vermis, and partial agenesis of the corpus callosum (Figure 3). Additional anomalies involved the absence of the anterior limb of the internal cells bilaterally, a dysplastic cerebellum (Figure 4), bilateral staphyloma and coloboma with extra-axial high T1 signal intensity (Figure 5), and a left parietal cephalohematoma. These findings collectively pointed toward a possible diagnosis of JSRD with elements of tubulinopathy. Tragically, the patient had a history of chronic lung disease, frequent episodes of desaturation, and bradycardia, ultimately resulting in a cardiac arrest. Despite extensive resuscitative efforts, no signs of recovery were observed, and the cause of death was primarily attributed to the intricate congenital anomalies and the profound brain malformations confirmed in the MRI findings, emphasizing the challenges posed by complex cases like this. 

## Discussion

Epidemiology

Data on the epidemiology of JS is scarce. To date, there has been no data regarding the incidence of the syndrome. Prevalence is estimated to be between 1/80,000 and 1/100,000 live births; however, these estimations are based on unpublished data [5].

Pathophysiology

Genetic mutations are the underlying causative factor for the manifestation of this syndrome. To date, more than 35 genes associated with JS have been identified [6]. It demonstrates both autosomal recessive and X-linked recessive modes of inheritance, with autosomal recessive inheritance being more frequently observed [7]. Sporadic cases have also been documented [8]. However, conflicting data persist regarding the prevalence of inherited versus sporadic cases. Amin et al. leaned toward the possibility of a sporadic pattern being the most common [9]. Conversely, Parisi suggested that the syndrome is predominantly inherited in the previously mentioned patterns [7]. Further research is warranted to thoroughly investigate the prevalence of inherited versus sporadic cases.

Symptomatology

JS manifests with a spectrum of symptoms involving multiple systems. The hallmark symptoms observed in most patients include hypotonia with subsequent ataxia and global developmental delay [5,10]. Hypotonia can manifest as early as in the neonatal period with poor head control and weak grasp reflex. As the child grows, it can affect the infant's ability to turn from a prone to a supine position or vice versa or the ability to sit upright without support at the expected age [11,12]. Subsequent ataxia develops in children who achieved independent walking [11], characterized by a wide-based, unsteady gait, with irregular steps, potentially affecting their ability to run or climb. Global developmental delay is usually severe and involves all components of developmental milestones, including motor, language, cognitive, and socio-behavioral milestones [13]. However, the severity is not related to the patient's age or severity of brain malformation on MRI [14]. Hodgkins et al. highlighted the challenge in evaluating cognitive delay, attributing the difficulty to the impairment of various developmental components essential for supplying necessary input crucial to cognitive processes. These components include gross and fine motor movements, ocular movements, and the inability to control speech. Consequently, their analysis led them to the conclusion that global developmental delay might generate a misleading impression of severe cognitive delay in the early stages of life [15].

Poretti et al. reported a 29-year-old woman with mild JS physical features and a normal IQ (94). After the syndrome was diagnosed in two of her siblings, screening for JS was conducted for her. The subsequent MRI revealed the characteristic molar tooth sign typical for JS, confirming the diagnosis in her case [16]. This case further supports the conclusion regarding the challenge of accurately assessing cognitive ability in individuals with JS, emphasizing the potential for overshadowing by other delayed developmental functions. These hallmark symptoms are usually accompanied by respiratory abnormalities in many patients. Breathing abnormalities may present as episodic tachypnea [17], central apnea [18], or alternating episodes of hyperpnea and apnea [19]. In some cases, it may be severe enough to cause respiratory failure that may require mechanical ventilation [20]. Other manifestations that could be seen in patients with JS include ocular symptoms and facial and body dysmorphism. In a review of ocular manifestations of JS conducted by Wang et al., results have shown that the most common ophthalmological symptom was ocular apraxia (80%), followed by strabismus (74%) and nystagmus (72%). Other ocular presentations included ptosis, Chori retinal coloboma, optic nerve atrophy, and retinal degeneration [21]. Other features associated with the syndrome include face/head and body dysmorphism and involvement of internal organs. Dysmorphic features of the face, head, and body present with a wide range of symptoms. Facial/head dysmorphism may include the following symptoms: frontal bossing, saddle or flat nose, protruded tongue, high-arched palate, teeth malocclusion, box-shaped head, polydactyly, simian crease, abnormal chest shape, wide-spaced nipples, abnormalities of the skeleton such as kyphoscoliosis or ribs agenesis, micropenis, and cryptorchidism [17,22,23]. Internal organs can be involved leading to deranged function of these organs, including liver and kidney diseases [24,25]. This is referred to as JSRD [26]. The endocrine system can also be involved. Acken et al. reported a case of a newborn presenting with features of JS, who was found to have multiple pituitary hormone deficiencies. Pituitary agenesis was seen on MRI [23].

Diagnosis

The diagnosis of JS mandates the presence of specific clinical manifestations and characteristic imaging findings. Employed imaging modalities for diagnostic purposes include CT and MRI of the head. A cranial CT scan may be employed to assess and visualize characteristic morphological attributes indicative of JS. This imaging modality is capable of revealing distinctive features such as hypoplasia affecting the postero-inferior sector of the cerebellar vermis. Furthermore, the CT scan may depict the presence of a dilated fourth ventricle and communication pathway connecting the fourth ventricle and the cisterna magna, often displaying varying degrees of cerebellar dysgenesis [27]. However, this imaging modality is less sensitive and reliable [28]. A crucial component of the diagnostic assessment for a child under suspicion of JS involves a high-quality MRI scan. This scan should encompass axial, coronal, and sagittal views, preferably with 3 mm axial planes through the midbrain and pons [28]. MRI findings in JS include a molar tooth sign (MTS), characterized by the underdevelopment of the cerebellar vermis, stemming from an unusually deep interpeduncular fossa and the presence of thickened and elongated cerebellar peduncles. This finding is characteristic of JS [29]. Other findings include abnormal thickening and orientation of bilateral superior cerebellar peduncles (SCP), midbrain atrophy, hypoplasia of superior vermis, prominent preoptic sheath, enlarged fourth ventricle (bat wing sign), microcephaly, large cerebellar hemisphere, inferior vermis hypoplasia, bulky medulla, hypoplasia of splenium, J-shaped sella, posterior fossa enlargement, and pons thinning [22,4,30]. Our patient’s MRI showed MTS, bat-wing sign, and incomplete absence of the corpus callosum isthmus and splenium.

The diagnosis of JS is contingent upon the presence of the following diagnostic criteria [10]. The MTS, a distinctive feature, is characterized by a deep interpeduncular fossa, prominent, straightened, and thickened superior cerebellar peduncles, and hypoplasia of the cerebellar vermis, particularly the midline region. High-quality MRI with thin (3-mm thickness) axial sections spanning from the midbrain to the pons, in addition to standard axial, coronal, and sagittal sections, is strongly recommended for its visualization [28]. Hypotonia in Infancy with subsequent onset of ataxia is another feature along with developmental delays / Intellectual disability.

Having either one or both of the subsequent clinical features, while not obligatory for diagnosis, can be considered as adjunctive indicators supporting the diagnostic evaluation: irregular breathing patterns during infancy, characterized by episodic apnea and/or tachypnea, which may at times alternate, and abnormal ocular movements, encompassing nystagmus and/or oculomotor apraxia (OMA) [31]. Conducting an ultrasound examination of the abdominal cavity is imperative in the diagnostic evaluation of JS. This comprehensive assessment encompasses the meticulous scrutiny of key organs, including the liver, and the kidneys, with the primary objective of identifying and assessing potential abnormalities. Given that these organ systems can be susceptible to involvement in JS, the ultrasound examination serves as a critical component of the diagnostic process, facilitating the early detection of any pathological alterations or anomalies [29].

Management

The management of JS involves conducting various evaluations following the initial diagnosis and addressing the manifestations of the syndrome in different body systems.

Evaluations done after the initial diagnosis include the following [28]: After the initial diagnosis, comprehensive evaluations are undertaken, encompassing a baseline neurological examination, including assessments of tone, eye movements, and cerebellar signs. In cases of symptomatic apnea, a baseline polysomnogram is recommended. Swallowing capabilities are assessed, along with an evaluation of developmental milestones based on the patient's age. An ophthalmological examination is conducted, and an abdominal ultrasound is performed. In addition, a thorough evaluation involves a complete blood count (CBC), renal and liver function tests, and urinalysis for specific gravity, assessing concentrating ability when feasible. For males with micropenis or any child displaying signs of growth hormone deficiency, an endocrine assessment is suggested to explore potential pituitary abnormalities. Suspected skeletal dysplasias, such as short-rib polydactyly or JS, prompt the need for a skeletal survey and/or limb radiographs. Consulting with a clinical geneticist is recommended to document family history, evaluate growth and head size, and examine for additional anomalies like polydactyly, dysmorphic facial features, tongue tumors/lobulations, and micropenis.

Treatment of manifestations

JS management is a multidisciplinary endeavor aimed at addressing manifestations across various body systems [28]. In the respiratory domain, infants and children with abnormal breathing patterns may undergo apnea monitoring, and supportive therapies like stimulatory medications or oxygen supplementation may be applied, especially in the newborn period. Anesthesia during surgical procedures for those with respiratory issues can be managed using regional anesthesia without opioids or alpha-2 agonists to avoid complications [22,32]. For hypotonia and related interventions, motor dysfunction can be managed by a speech therapist, and feeding solutions like nasogastric or gastrostomy tubes may be employed for severe dysphagia. Early intervention programs involving occupational, physical, and speech therapy are recommended, along with individualized educational assessments for school-aged children. In addressing other CNS malformations, neurosurgical consultation is advised for evidence of hydrocephalus, while posterior fossa cysts rarely require intervention. Seizures should be evaluated and treated by a neurologist, with psychotropic medications considered for behavioral complications. Ophthalmologic interventions may include surgery for symptomatic issues, corrective lenses, and possible vision therapies. Renal disease management involves consultation with a nephrologist, and in cases of end-stage renal disease (ESRD), dialysis or kidney transplantation may be necessary. Hepatic fibrosis requires consultation with a gastroenterologist, and surgical interventions such as portal shunting may be considered. Skeletal issues like polydactyly may require surgical treatment, and scoliosis can be managed by an orthopedic specialist.

Other interventions include standard surgical procedures for orofacial clefting, surgical resection for tongue tumors, and evaluation for obstructive sleep apnea. Consultation with an endocrinologist is recommended for menstrual irregularities and pituitary hormone deficiency. Obesity should be managed through appropriate measures, and congenital heart defects or situs abnormalities should be treated conventionally. Surgical correction of Hirschsprung disease is indicated if present [28] (Table 1).

**Table 1:** Summary of the discussion.

Reference	Year	Sex	Age (years old)	Clinical presentation	Imaging and radiographic features	Differential diagnosis	Treatment and prognosis
Amin A [8]	2011	M	NA	Episodes of breathlessness and developmental delay	MRI (molar tooth sign)	Joubert’s syndrome	NA
Amin OS [9]	2010	M	6	Ataxic cerebral palsy then developed generalized tonic-clonic seizures	MRI (molar tooth sign)	Joubert’s syndrome	Generalized seizures were controlled with Lamotrigine
Boltshauser E [11]	1976	F	6 months	Intermittent shallow respirations at a rate up to 180/min and developmental delay	Pneumoencephalography (enlarged and unusually shaped fourth ventricle, suggesting partial agenesis of the cerebellar vermis, particularly of its superior part)	Autosomal inherited syndrome	No progress
Boltshauser E [11]	1976	M	Neonate	Tachypnea interrupted by apneic spells and developmental delay	Pneumoencephalography (lateral ventricles were not significantly dilated, but the fourth ventricle was markedly dilated and there was an extensive accumulation of air in the cisterna magna)	NA	Death at 2 years and 9 months due to pneumonia
Boltshauser E [11]	1976	M	Neonate	Cyanosis, shallow respiration and several apneic spells, delayed psychomotor development	Pneumoencephalography (a slightly dilated fourth ventricle which was also markedly deformed in its upper part, suggesting dysplasia primarily of the superior cerebellar vermis)	NA	Slight improvement in his gait with progressive scoliosis and he never spoke
Poretti A [16]	2010	F	20	Minimal truncal ataxia and oculomotor apraxia	MRI (molar tooth sign)	Joubert’s syndrome	NA
Fluss J [17]	2015	M	Fetus	Fetal ultrasound finding of a prominent cisterna magna	MRI (dysgenesis of the vermis and a molar tooth sign)	Joubert’s syndrome	NA
Taytard J [18]	2020	F	12	Central sleep apnea with transient episodes of tachypnea	MRI (cerebellar vermis dysplasia and hypoplasia without the characteristic appearance of the midbrain MTS on axial images)	Cerebellar malformation	Successfully treated with bilevel positive airway pressure
Barzegar M [19]	2013	F	8 months	Seizure	MRI (vermis agenesis and molar tooth sign)	Joubert’s syndrome	NA
Barzegar M [19]	2013	M	2.3	Severe global developmental delay	MRI (vermis agenesis and molar tooth sign)	Joubert’s syndrome	NA
Akcan N [23]	2019	M	13 days	Hypoglycemia and micropenis	MRI (molar tooth sign and pituitary agenesis)	Joubert’s syndrome	Steroid and then L-thyroxine replacement treatment GH treatment was started at 1.5 years of age. His growth rate is controlled every 6 months
Dekaban AS [25]	1969	M	Neonate	Poor vigor and convulsions	Pneumoencephalogram revealed slight symmetrical dilation of all ventricles	NA	Death at 3 years
Dekaban AS [25]	1969	F	Neonate	Small lump on the back of her head, Oscillatory eye movement	X-ray (rounded defect in the occipital bone), pneumoencephalography (dilated ventricles and communication between the ventricles and the occipital meningocele, the fourth ventricle and the cisterna magna were considerably enlarged, hypoplasia of the cerebellum)	NA	Death at 4 years
Jain D [27]	2022	F	21	History of delayed milestones, infantile hemiplegia with hearing and visual impairment	History of delayed milestones, infantile hemiplegia with hearing and visual impairment	Joubert’s syndrome	NA
Zhu F [30]	2019	M	16	Sudden pale face and chest pain for half-an-hour (congenital cataract in the right eye, as well as nystagmus and generalized hypotonia)	MRI (cerebrospinal fluid line between the bilateral cerebellar hemispheres, enlarged fourth ventricle with abnormality in shape and bilaterally elongated superior cerebellar peduncles with deep interpeduncular fossa)	Joubert’s syndrome	NA
Andermann F [4]	1999	M	6 months	Developmental delay and abnormal respiratory pattern	Reported imaging findings that highlighted the core abnormalities: agenesis of the cerebellar vermis, abnormal 4th ventricle, and thin pons. MRI (molar tooth sign)	Joubert’s syndrome	The improvement in balance, respiratory abnormality, and oculomotor disturbance over the years is quite striking, although deficits in these areas are still apparent. His intellectual function approaches the borderline range, and his major current disability is in the area of social judgment.
Vodopich DJ [32]	2004	M	7 months	Elective right inguinal hernia repair	Reported to show abnormal, periodic breathing with tachypnea followed by apnea, including one severe apneic event leading to cardiac arrest (MRI) (agenesis of corpus callosum, large posterior fossa cyst with dysplastic inferior cerebellar vermis, and buckled-appearing anterior margin of the pons)	Joubert’s syndrome	NA
Our case	2023	NA	88 days	Multiple congenital anomalies	MRI (thickened elongated superior cerebellar peduncles, an abnormal midbrain-to-pons ratio, a characteristic "batwing" appearance of the fourth ventricle, aplastic cerebellar vermis, and partial agenesis of the corpus callosum)	Joubert’s syndrome	No signs of recovery were observed, and the cause of death was primarily attributed to the intricate congenital anomalies and the profound brain malformation

## Conclusions

The case report highlights the diagnostic challenges of JSRD due to their diverse clinical manifestations. The importance of neuroimaging, particularly MRI and CT scans, is emphasized for identifying the "molar tooth sign," a key feature in diagnosing JSRD. The report reviews pediatric cases, focusing on diagnostic imaging features. JS, an autosomal recessive disorder, presents with cerebellar vermal agenesis, ataxia, developmental delays, and other neurological symptoms. The characteristic imaging findings include cerebellar abnormalities and a distinct molar tooth pattern, which are crucial for accurate diagnosis. Treatment involves a multidisciplinary approach to manage the various symptoms associated with the disorder.

## References

[1] Joubert M, Eisenring JJ, Andermann F (1968). Familial dysgenesis of the vermis: a syndrome of hyperventilation, abnormal eye movements and retardation. Neurology.

[2] Barkovich A (2005). Pediatric neuroimaging. https://www.ajnr.org/content/28/1/192.

[3] Parisi M, Glass I (2003). Joubert syndrome. Neurology.

[4] Andermann F, Andermann E, Ptito A, Fontaine S, Joubert M (1999). History of Joubert syndrome and a 30-year follow-up of the original proband. J Child Neurol.

[5] Valente EM, Brancati F, Boltshauser E, Dallapiccola B (2013). Clinical utility gene card for: Joubert syndrome--update 2013. Eur J Hum Genet.

[6] Spahiu L, Behluli E, Grajçevci-Uka V, Liehr T, Temaj G (2022). Joubert syndrome: molecular basis and treatment. J Mother Child.

[7] Parisi MA (2019). The molecular genetics of Joubert syndrome and related ciliopathies: The challenges of genetic and phenotypic heterogeneity. Transl Sci Rare Dis.

[8] Amin A (2011). Joubert’s syndrome: a case report. J Nepal Paedtr Soc.

[9] Amin OS, Shwani SS (2010). Ataxia, hyperpnoea and mental retardation: was it the molar tooth?. BMJ Case Rep.

[10] Buke B, Canverenler E, İpek G, Canverenler S, Akkaya H (2017). Diagnosis of Joubert syndrome via ultrasonography. J Med Ultrason (2001).

[11] Boltshauser E, Isler W (1977). Joubert syndrome: episodic hyperpnea, abnormal eye movements, retardation and ataxia, associated with dysplasia of the cerebellar vermis. Neuropadiatrie.

[12] Joubert M, Eisenring JJ, Robb JP, Andermann F (1969). Familial agenesis of the cerebellar vermis. A syndrome of episodic hyperpnea, abnormal eye movements, ataxia, and retardation. Neurology.

[13] Kozina AA, Kanaeva GK, Baryshnikova NV (2023). A case of Joubert syndrome caused by novel compound heterozygous variants in the TMEM67 gene. J Int Med Res.

[14] Gitten J, Dede D, Fennell E, Quisling R, Maria BL (1998). Neurobehavioral development in Joubert syndrome. J Child Neurol.

[15] Hodgkins PR, Harris CM, Shawkat FS (2004). Joubert syndrome: long-term follow-up. Dev Med Child Neurol.

[16] Poretti A, Dietrich Alber F, Brancati F, Dallapiccola B, Valente EM, Boltshauser E (2009). Normal cognitive functions in joubert syndrome. Neuropediatrics.

[17] Fluss J, Blaser S, Chitayat D, Akoury H, Glanc P, Skidmore M, Raybaud C (2006). Molar tooth sign in fetal brain magnetic resonance imaging leading to the prenatal diagnosis of Joubert syndrome and related disorders. J Child Neurol.

[18] Taytard J, Valence S, Sileo C (2020). Severe central apnea secondary to cerebellar dysplasia in a child: look past Joubert syndrome. J Clin Sleep Med.

[19] Barzegar M, Malaki M, Sadegi-Hokmabadi E (2013). Joubert syndrome with variable features: presentation of two cases. Iran J Child Neurol.

[20] Mercado A, Pedraza L, Mayol PM, Rodríguez Santana R, Tejeda C (1991). Joubert syndrome. Respiratory failure requiring home mechanical ventilation. Bol Asoc Med P R.

[21] Wang SF, Kowal TJ, Ning K, Koo EB, Wu AY, Mahajan VB, Sun Y (2018). Review of ocular manifestations of Joubert syndrome. Genes (Basel).

[22] Sriganesh K, Vinay B, Jena S, Sudhir V, Saini J, Umamaheswara Rao GS (2014). Anesthetic management of patients with Joubert syndrome: a retrospective analysis of a single-institutional case series. Paediatr Anaesth.

[23] Akcan N, Bas F, Poyrazoglu S, Bundak R (2019). Joubert syndrome with multiple pituitary hormone deficiency. BMJ Case Rep.

[24] Strongin A, Heller T, Doherty D (2018). Characteristics of liver disease in 100 individuals with Joubert syndrome prospectively evaluated at a single center. J Pediatr Gastroenterol Nutr.

[25] Dekaban A (1969). Hereditary syndrome of congenital retinal blindness (Leber), polycystic kidneys and maldevelopment of the brain. Am J Ophthalmol.

[26] Brancati F, Dallapiccola B, Valente EM (2010). Joubert Syndrome and related disorders. Orphanet J Rare Dis.

[27] Jain D, Ravishankar V (2022). Ocular manifestations leading to a diagnosis of Joubert syndrome related disorder. Nepal J Ophthalmol.

[28] Parisi MA, Doherty D, Chance PF, Glass IA (2007). Joubert syndrome (and related disorders) (OMIM 213300). Eur J Hum Genet.

[29] Quisling RG, Barkovich AJ, Maria BL (1999). Magnetic resonance imaging features and classification of central nervous system malformations in Joubert syndrome. J Child Neurol.

[30] Zhu F, Wang Y, Yang G, Deng S (2019). Brain magnetic resonance imaging of Joubert syndrome: case presentation in a child. Quant Imaging Med Surg.

[31] Parisi MA (2009). Clinical and molecular features of Joubert syndrome and related disorders. Am J Med Genet C Semin Med Genet.

[32] Vodopich DJ, Gordon GJ (2004). Anesthetic management in Joubert syndrome. Paediatr Anaesth.

